# Global Burden of Lumpy Skin Disease, Outbreaks, and Future Challenges

**DOI:** 10.3390/v15091861

**Published:** 2023-08-31

**Authors:** Mahfuza Akther, Syeda Hasina Akter, Subir Sarker, Joshua W. Aleri, Henry Annandale, Sam Abraham, Jasim M. Uddin

**Affiliations:** 1 Department of Pathology and Parasitology, Faculty of Veterinary and Animal Science, Hajee Mohammad Danesh Science and Technology University, Dinajpur 5200, Bangladesh; mahfuzaakther@hstu.ac.bd; 2 Faculty of Veterinary Science, Bangladesh Agricultural University, Mymensingh 2202, Bangladesh; hasina.akter@murdoch.edu.au; 3 School of Veterinary Medicine, Murdoch University, Perth, WA 6150, Australia; j.aleri@murdoch.edu.au (J.W.A.); henry.annandale@murdoch.edu.au (H.A.); 4 Biomedical Sciences & Molecular Biology, College of Public Health, Medical and Veterinary Sciences, James Cook University, Townsville, QLD 4814, Australia; subir.sarker@jcu.edu.au; 5 Centre for Biosecurity and One Health, Harry Butler Institute, Murdoch University, Perth, WA 6150, Australia; s.abraham@murdoch.edu.au

**Keywords:** economic impact, global biosecurity challenges, historical outbreak, lumpy skin disease, vaccination

## Abstract

Lumpy skin disease (LSD), a current global concern, causes economic devastation in livestock industries, with cattle and water buffalo reported to have higher morbidity and lower mortality rates. LSD is caused by lumpy skin disease virus (LSDV), a member of the Poxviridae family. It is an enzootic, rapidly explorative and sometimes fatal infection, characterized by multiple raised nodules on the skin of infected animals. It was first reported in Zambia in 1929 and is considered endemic in Africa south of the Sahara desert. It has gradually spread beyond Africa into the Middle East, with periodic occurrences in Asian and East European countries. Recently, it has been spreading in most Asian countries including far East Asia and threatens incursion to LSD-free countries. Rapid and accurate diagnostic capabilities, virus identification, vaccine development, vector control, regional and international collaborations and effective biosecurity policies are important for the control, prevention, and eradication of LSD infections. This review critically evaluates the global burden of LSD, the chronological historical outbreaks of LSD, and future directions for collaborative global actions.

## 1. Background

The increasing emergence or recurrence of major transboundary and emerging animal diseases in recent decades has become a matter of great economic and public health concern worldwide. These diseases affect food security by reducing the availability and affordability of high-quality animal products [1]. In recent years, lumpy skin disease (LSD) has been identified as one of the most devastating and emerging threats to large domesticated ruminants such as cattle, water buffalo and wild bovine species [2,3]. The World Organization for Animal Health (WOAH) has listed LSD as one of the most economically important and notifiable trans-boundary viral animal diseases. The disease is also known as pseudo-urticaria, Neethling pox virus disease, Exanthema nodularis bovis and Knopvelsiekte [4]. LSD is caused by lumpy skin disease virus (LSDV), a member of the genus *Capripoxvirus* within the Family Poxviridae. LSDV is antigenically closely related to sheep and goat poxviruses but differs from them phylogenetically [5,6].

LSD was reported for the very first time in 1929 (in Zambia) and, since then, was considered to be confined to various areas of Africa, where periodic outbreaks would be recorded until 1986 [7]. The first occurrence of LSD outside of Africa was reported in Israel between 1986 and 1988 and has gradually spread to the Middle East, then Eastern Europe and Russia [8], with subsequent spread through the Balkans [9]. In 2019, new cases were reported in South and East Asia [2], presenting an ongoing threat to all the Indo-Asian countries including Afghanistan, Pakistan and India [10,11]. Current active case identification in Asia (China, Cambodia, Singapore and Indonesia) has raised concerns about the intrusion of this virus into LSD-free countries with large, naïve cattle populations, such as Australia [12].

This contagious disease is transmitted by a variety of vectors such as biting flies, lice, ticks, mosquitoes, and wasps, but also through close contact with infected animals or contaminated feed and water troughs [5,13]. Hot and humid weather is responsible for vector multiplication and an increase in activity, making the rainy summer and autumn season and low marshy land epidemiologically more suitable for the occurrence of this disease [9,14].

LSD is characterized by high morbidity and low mortality rates and, depending on the host immune response, affected animals can display acute or chronic clinical disease [13]. Fever, anorexia, lymphadenopathy, rhinorrhea and distinct skin lesions define the acute infection stage, whereas animals in the chronic infection stage display poor production and infertility. The economic impact of LSD is realized though a drop in milk production, poor-quality hides and meat, abortion and death [15,16,17].

Preventing the spread of LSD to a disease-free area or stamping out of the disease in an area would require several control measures including strict quarantine, restriction of animal movement, vaccination with live attenuated vaccines (either homologous or heterologous or both), isolation and slaughter of affected animals, proper disposal of carcasses, cleaning and disinfection of the premises and, very importantly, insect control [14,18,19]. Occasionally, whole herd depopulation has been recommended, but in endemic scenarios, the affected farms often isolate sick animals and provide supportive treatment that may include wound dressings to prevent fly infestations and secondary infections [18,19,20].

The emergence pattern, re-emergence record and risk of incursion of LSD to uninfected countries are gradually increasing, and therefore the importance of identification of new measures which could be beneficial for rapid tracing of the infection and formation of definite control strategies takes on greater urgency. Genomic information is a vital resource that has recently been used for quick tracing, typing and identification of the mutation point of infectious agents and to develop appropriate control and eradication strategies [21,22]. This review, therefore, also provides a perspective on the use of the genetic basis of LSDV to establish convenient diagnostic and control measures.

## 2. Virus, Pathology and Transmission

### 2.1. Lumpy Skin Disease Virus

Lumpy skin disease is a WOAH-marked highly contagious vector-borne emerging transboundary pox-viral infection of bovine species [23,24]. The disease is caused by lumpy skin disease virus (LSDV), which belongs to the genus Capripoxvirus (CaPV) under the subfamily of Chordopoxvirinae within the family of Poxviridae [22]. The genus capripoxvirus is comprised of sheep pox virus (SPPV), goat pox virus (GTPV) and lumpy skin disease virus (LSDV) and they all bear approximately 96% vaccine cross-protection [22,25,26,27].

### 2.2. Viral Structure, Nature, and Genome Characteristics

LSDV is an oval- or brick-shaped virus with a length of 294 ± 20 nm and width of 262 ± 22 nm with bilateral body and covered by bilipid bilayer. The lineage of LSDV is reported as Viruses > Varidnaviria > Bamfordvirae > Nucleocytoviricota > Pokkesviricetes > Chitovirales > Poxviridae > Chordopoxvirinae > Capripoxvirus. This virus ports the double-stranded, linear DNA genome of 151 kbp in size. The central region codes for chordopoxvirinae conserve, open reading frames (ORFs) annotated as putative gene coding for polypeptides of 53 to 2025 amino acids. The LSDV genome consists of a central coding region bounded by identical 2.4 kbp-inverted terminal repeats and contains 156 putative genes. Among them are 146 conserved genes which encode proteins involved in transcription and mRNA biogenesis, nucleotide metabolism, DNA replication, protein processing, virion structure and assembly, and viral virulence and host range [22,27]. LSDV has an additional nine genes specifically adapted for cattle infection and which are noted to be inactive in SPPV and GTPV [28]. Although most of the genes are identical between members of CaPV, the variable genes named G-protein-coupled chemokine receptor (GPCR) gene is used for genetic differentiation among them [29]. These genes are noted as stable in the past, but gradually a high frequency of synonymous mutations by natural drift and non-synonymous mutations with highly cell passaged viruses become a subject of concern. The alterations are recorded and submitted by different researchers and stored in Genbank, National Center for Biotechnology Information (NCBI). Hence, genomic study using high-throughput sequencing is important to understand the host–pathogen interactions. Sequencing technology has become an important tool for accurate detection and characterization of specific mutations or genes responsible for pathogenicity, immune evasion, vaccine escape, recombination or reassortment, virulence, transmissibility, tissue trophism and replication factors of LSDV [21,30,31]. The genome sequence of LSDV can produce information about its geographical origins, spatio-temporal spreading and disease pattern, re-emergence and the nature of infection. The identification of specific markers may be used for contact tracing, to identify vaccine candidates, and virus control and prevention policies. The identification of alteration or deletion of a specific marker responsible for viral replication and pathogenicity could be used for vaccine production [31,32,33]. In addition, the mutation pattern of genomes can be used to uncover potential outbreaks and interlink the existing unrelated outbreaks [30]. Hence, there are possibilities of using high-throughput sequencing (HTS) for outbreak prognosis and defending biosecurity threats.

### 2.3. Viral Replication and Resistance

Similar to other members of the poxviridae family, LSDV exhibits a cytoplasmic replication cycle, where the double-stranded DNA (dsDNA) is largely enzymatically mediated for both messenger RNA (mRNA) production and genome copying for progeny virions [9,34]. Actual viral DNA synthesis begins 1.5 to 6 h after infection and two types of infectious virions (single membranous and double membranous) are released from the infected cells [35]. Recent studies on the susceptibility and resistance of LSDV describe the virus as greatly susceptible to a wide range of temperature (55–65 °C) and pH (6.6–8.6) variation and resistant to a wide range of physical and chemical components. It is resistant to inactivation and can remain viable for up to 35 days in desiccated skin crusts, for >33 days in skin necrotic nodules and for at least 18 days in air-dried hides [13,36]. It may persist in the environment for longer periods of time, especially in dark conditions in contaminated animal sheds, where it can persists for several months [37]. This persistent nature makes LSDV an important biosecurity threat for global livestock industries.

### 2.4. Host–Pathogen Interaction

Lumpy skin disease virus is highly host-specific and causes disease only in bovine species such as cattle and water buffalo [38,39], whereas the other domesticated species such as sheep, goats, pigs and horses are not affected due to host specificity [40,41]. Cattle are the definitive hosts but specific antibodies for LSDV have been found in various wild ruminants such as blue wildebeest, eland, giraffe, impala and greater kudu, whenever experimental inoculation was carried out [42,43,44]. However, it is necessary to observe and monitor disease outbreaks and virus mutations regularly to anticipate potential host jump.

The degree of severity of LSD in hosts depends upon several host and environmental factors [45]. Although breed, age and sex variation do not seem to play a role in determining the severity of disease [13,39,46], some studies found *Bos taurus* more susceptible to severe disease than indigenous (*Bos indicus*) and Zebu cattle [47] due to their thin-skin characteristics and also high-producing milk production [48]. Male zebu cattle often become susceptible due to working as draught animals, resulting in skin scratches which become the site of vector attraction [6]. Calves tend to display more severe clinical signs than adults [6,49].

Under ideal environmental conditions, vector populations may proliferate and come into more regular contact with susceptible hosts. Environmental and land use policies therefore play an important role in management of vector-borne diseases such as LSD. Farming in low land with continuous watercourses provides a humid condition relatively suitable for the higher proliferation of these vectors [2,6,14]. The farming system designed for high yielding cows combined with favorable environmental condition could create the stressful condition, which may provoke host immunosuppression and production of disease [50]. Comparatively larger farms with intensive housing, shared feeding and watering facilities may also help in spread of LSDV [51].

Due to the remarkable stability of the virus in different infective sites and environmental conditions, with better resistance against most of the physical and chemical inactivators, LSDV can persist in skin lesions for up to 35 days at ambient temperature [52]. The virus can be shed through the nasal and lacrimal secretions, milk and semen, and are noted to be viable for up to 11 days in milk and 22 days in semen [53,54]. There is no evidence of virus presence in the meat from infected animals but the virus can be isolated from infected fomites including animal rearing and transportation equipment [55]. There is a lack of evidence of viral presence in the vector body after 4 days of inoculation [49].

The host immune response is not clearly understood but, as with most of the capripox viruses, a lifelong immunity against reinfection with LSDV has been demonstrated [56]. After infection, the growth and replication of LSDV occurs intracellularly [57,58], so the humoral immunity cannot resist the viral proliferation but innate immunity has the capacity to mount an immune response by stimulating the adaptive immune system and mediating several immune cells such as lymphocytes, macrophages and neutrophils, responsible for inactivating the causal agent and controlling the disease [28,56,59]. Animals that have recovered from natural infection produce specific antibodies, capable of neutralizing up to 3 logs of the virus and are also resistant to re-infection [52]. The maternal immunity may support calves to resist the clinical infection of LSD for up to 6 months [60]. Vaccination can stimulate the humoral immunity of the animal and give protection for more than 7 months [59], so an annual vaccination schedule with a booster dose is recommended due to the unknown duration of both cellular and humoral immunity [61]. However, LSDV–host interaction remains unclear at this time.

### 2.5. Viral Transmission

In general, LSDV is thought to be transmitted by the indirect route, with a blood-sucking arthropod as the vector [62]. The vector associated with transmission transmits the viral particle through its mouth parts without any viral replication in the host itself, hence this transmission is considered mechanical, instead of biological [6]. Some biting flies such as Stable flies (*Stomoxy calictrans*) and biting files (*Biomyia fasciata*), mosquitoes such as *Culex mirificens* and female *Aedes egypti* and the biting midge, *Culicoides nubeculosus,* are commonly involved in the transmission of LSDV [43,63]. Additionally, house flies (*Musca domestica*) and tsetse flies (*Glossina Sp.*) may also play a role in LSDV transmission [64]. Several hard ticks (*Amblyomma hebraeum*, *Rhipicephalus appendiculatus* and *Rhipicephalus decoloratus*) may also serve as reservoirs as well as mechanical vectors for LSDV [64]. Recent studies have shown that *Boophilus decoloratus* is one of the tick species which can transmit LSDV by both transstadial and transovarian means [63,65]. However, the vector availability and distribution vary in different geographical and climatic regions, so further detailed studies in other geographical areas are warranted.

**Figure 1:**
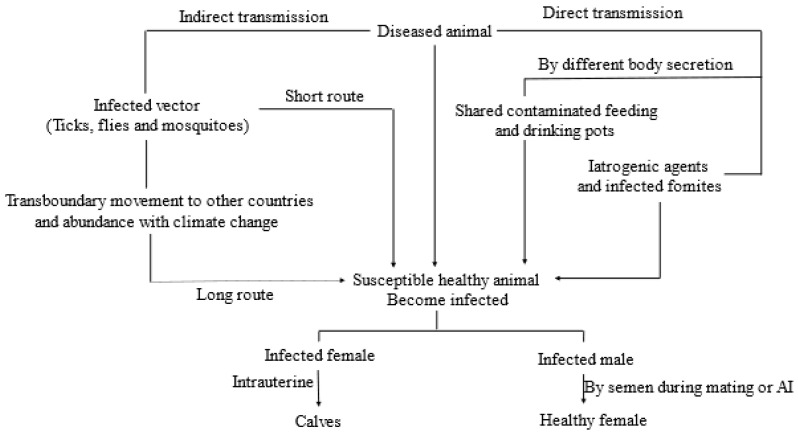
Schematic representation of the possible mode of transmission of LSDV.

Direct contact with infected animals has been shown to be an effective method of LSDV transmission [66,67] since they shed the virus through skin lesions, nasal, oral and ocular secretions, and from lesions containing mucosa [68]. Direct transmission of this disease then occurs through the introduction of shared feeding and watering pots contaminated with these body secretions. It can also be transmitted iatrogenically during mass treatment using common treatment tools such as needles [6,55,69]. Lactating cows can also transmit LSDV to suckling calves by milk contaminated with udder skin lesions, while vertical transmission has been demonstrated experimentally through the transmission of infected semen during natural mating or artificial insemination; the virus may remain persistent for up to 42 days post-infection in semen [70]. An intrauterine route of infection was recorded as vertical transmission from dam to calves [68,71] and it is assumed that this virus can be shed through vaginal secretion [48,72]. Direct transmission accounts for short route infection, whereas indirect or vector borne transmission can be applicable for both short and long-distance infection followed by uncontrolled transboundary animal movement [45,73,74]. A representation of the possible mode of transmission of LSDV is shown in Figure 1.

### 2.6. Pathogenesis and Effects on Host Body

There is limited knowledge on the pathogenesis of LSD [71,75] compared to other viral diseases. LSD shows a progressive pattern of pathogenesis from viral inoculation followed by implantation and multiplication locally, then development of viremia resulting in viral transportation to the specific tissues and organs [57,76]. The incubation period of LSDV is approximately 5 weeks in the case of natural infections, while it ranges from 4 to 7 days experimentally [13]. Similar to the other members of Capripoxvirus, LSDV has a tissue trophism for keratinocytes [77]. Just after inoculation into the susceptible animals, either naturally or experimentally, the virus starts to replicate in most abundant cells such as keratinocytes, hair follicle epithelium, fibroblasts, interstitial macrophages and pericytes of the predilection site such as the skin of the head, neck, genitalia, limb and udder [76]. In keratinocytes, initially hyperplasia and ballooning degeneration occur, which may extend to the epidermis and several micro-vesicles form, then, larger vesicles form by the coalescence of micro-vesicles and attract the inflammatory cells to accumulate in the epidermis, dermis and subcutaneous layer [78]. Finally, there is ulceration with exudation resulting in scab and crust formation with different degrees of hemorrhage, congestion and edema formation in the surroundings [9,49]. The viral particles may be transported to secondary sites of infection including lungs, liver, kidney and other lymph-nodes [75,79] through systemic circulation; especially monocytes are considered as the carrier of LSDV and intermittent fever is the indication of extreme viremia. During virama there may be the possibility of the development of vasculitis and lymphangitis resulting from endothelial injury with viral replication [80].

### 2.7. Clinical Manifestation

Depending on the lump count, vector load, host susceptibility and immunity, managerial and environmental factors, clinical manifestations of LSD can be divided into mild and acute forms and starts with biphasic fever, rise to a peak of 40–40.5 °C, which can persist for 3 days [9,81]. In the mild form, very few number of nodular lesions of 1–5 cm in diameter are found within 2–3 days after the onset of fever along with anorexia, depression, hypersalivation, nasal and ocular secretion, decreased production and emaciation. The nodules are usually round, raised, hard, painful and hyperemic and usually observed in the skin of the muzzle, neck, back, legs, scrotum, perineum, eyelids, lower ear, nasolacrimal mucosa, and tail [72]. The acute form is more severe, with continuous high pyrexia, severe anorexia and depression, often many uniform nodules may be observed over the animal body within 2–3 weeks of onset of the disease. The raised, nodular lesions are usually approximately 1–7 cm in size and most commonly found in the head, neck, udder, genitalia, perineum and legs and can easily be separated from the surrounding healthy skin by a hemorrhagic rim [81]. The smaller nodules often coalesce to form larger lesions and slough off creating a condition named as ‘sit-fasts’, which serve as the nidus for further vector attraction and secondary bacterial infection [78,82]. Due to involvement of the whole skin and muscles, severe pain results in the animal becoming reluctant to move; occasionally swelling of the face, brisket and limb are also observed [54,83]. Affected animals also exhibit some typical LSD lesions in the oral cavity, conjunctiva and nasal cavity resulting in profuse mucosal secretion [39,50]. In affected cows, severe loss of milk production, abortion and persistent anestrous may develop, whereas, in male animals, due to testicular tissue involvement, clinical swelling of the scrotum is observed, which may lead to temporary or permanent infertility [39,72].

### 2.8. Hematological Assessment

The hemogram analysis shows no significant alteration in the blood profile in the early (within 1–2 days) stage of infection but as the disease progresses, several alterations are noted in the erythrogram, leucogram and others [84]. In prolonged cases, a marked decrease in total erythrocyte count (TEC), hematocrit value (HCT), hemoglobin value (Hb) and mean corpuscular hemoglobin concentration (MCHC) with an increased mean corpuscular volume (MCV) [85] may indicate the development of hemorrhagic or hemolytic anemia. A macrocytic hypochromic type [86] of anemia may develop due to several inflammatory cytokines (TNF, IL-1α, IL-1β, and IF-γ), initiating reduced erythropoiesis by the bone marrow. This type of anemia develops slowly and is usually mild in nature [84] and associated with anorexia, dietary iron deficiency or disturbed iron metabolism [87]. In acute infections, a large amount of endogenous corticosteroid is produced which leads to lymphopenia because of elevated viral load and leukopenia due to increased tissue demand and neutrophilic margination [88,89]. After 10–14 days of infection, leukocytosis (granulocytic) may occur, associated with the marked production and accumulation of neutrophils due to secondary pyogenic bacterial infection [88,89,90]. The platelet count may decrease leading to a marked thrombocytopenia, with a resultant short life span and extreme consumption by vasculitis and vascular thrombus formation [91].

### 2.9. Biochemical Assessment

The serum biochemical analysis is associated with alterations in the protein, creatinine, minerals and enzymatic level. Total serum protein and albumin are decreased during early LSDV infection, due to decreased dietary protein intake and reduced protein synthesis as a result of hepatocellular disturbances with higher protein catabolism [92]. Elevated globulin levels and blood urea nitrogen (BUN) are detected during the later stage of infection, which may be due to dehydration and the host immune response [91,93,94]. Elevated serum alanine amino transferase (ALT), aspartate aminotransferase (AST) and alkaline phosphatase (ALP) in early infection originate primarily from hepatocytic damage by the virus directly [92]. Raised AST activity with cardiac troponin activity confirms the cardiac tissue effect of LSDV [95,96]; however, skeletal muscle and myocardial damage are also indicated by increased serum lactate dehydrogenase (LDH) and creatine phosphokinase (CPK) activity [28,97,98]. Total blood glucose level and serum creatinine levels are also increased in infected cattle due to higher glucose catabolism in anorexia and kidney damage, respectively [89].

### 2.10. Pathological Assessment

In sum, there are skin nodules of uniform size, small, raised with erected hair, often merging to larger, irregular ones, spread all over the body surface [9]. These nodules are commonly present in the epidermal and dermal layer but subcutis and often deep musculature are also involved [7,99]. The appearance of the nodular lesions is associated with the progression of disease with definite “sit-fast” lesion characterized by severe ulcerative and suppurative necrosis with external scab or crust formation in acute cases. The necrotic lesion can eventually be found in almost all the parts of alimentary (lips, gum, palate, fore stomach), respiratory (nasal mucosa, pharynx, larynx, trachea and bronchiolar structures) and urogenital tract of both male (testes) and female (uterus, vagina, teats and udder) animals. In severe infection, the deeper tissues such as the tendon sheaths, synovial joints and even muzzle bone are also be affected [13]. Both local and mediastinal lymph nodes enlarge severely due to edema [100]. Pneumonic lung, tracheal dysfunction, mastitis, metritis, pyometra, and orchitis are common complications found, whenever there is secondary bacterial or fungal involvement [69,101].

Histopathological observation of LSD displays the pathognomonic microscopic lesion of eosinophilic intracytoplasmic inclusion bodies found in keratinocytes, macrophages, endothelial cells and pericytes from skin nodules [78,102]. There is marked ballooning degeneration of the cellular layer of both the epidermis and dermis which extend to macrovesicle formation with accumulation of inflammatory exudates [103,104]. The presence of marked inflammatory cells in the infection sites including the lymphocytes (for viral infection), macrophages (for phagocytosis), eosinophils (for inflammation), and neutrophil in the case of secondary infection are reported [68]. Similar lesions extend towards the subcutis and Zenker’s necrosis is noted when there is involvement of subcutaneous muscular structure [52]. There is marked congested blood vessels and edema, accompanied by marked accumulation of epithelioid cells in the dermal stoma [14]. There is severe vasculitis characterized by thickened vascular walls with inflammatory cell accumulation which finally leads to thrombus formation and concurrent necrosis [105,106]. The microscopic lesions are remarkably similar throughout the body [107].

## 3. Historical Outbreaks and Re-Emergence

Historically, LSD was first reported in Northern Rhodesia (now Zambia) in 1929 (MacDonald, 1931) as a condition of either poisoning or insect bite hypersensitivity, known as ‘pseudo-urticaria’ [108]. The infective nature of this condition was demonstrated between 1943 and 1945, when Zimbabwe (Southern Rhodesia), Botswana, and the Republic of South Africa reported cases [100,109]. By 1946, it had extended eastwards to Mozambique [46], then to central African countries such as Angola and Zaire in 1950, Madagascar in 1954 and finally Namibia, Tanzania and Uganda in 1956 [69]. Over the next two decades research of this infection was undertaken in different African countries. LSD first emerged in Kenya in 1957 and another re-emergence was recorded in 1977 [108,110]. After a gap of 14 years, another African country, Sudan, reported an explosive epizootic emergence of LSD in 1971 [111,112]. Chad and Niger experienced introduction of LSD in 1973 and were largely responsible for the outbreak in Nigeria in 1974 [113,114]. In Ethiopia, LSD was first observed in 1983 [115] and it gradually spread to almost all regions and agroecological zones of this country [49]. Several sporadic occurrences of LSD in Tanzania, Kenya, Zimbabwe, Somalia and Cameroon were recorded in different studies in the period of 1950 to 1985 with spontaneous re-emergence in different African countries over that timeline [8,116].

Outside the Sub-Saharan continent, an LSD outbreak was first reported in 1988 in Egypt, followed by Israel in 1989 [44,69]. Although a smaller outbreak with cases not being confirmed, LSD was reported in Oman in 1984 with an epizootic re-emergence in 2009 [50]. The disease was for the first time detected in Kuwait in 1986 and again in 1991. Furthermore, prevalence of LSD for the first time was recoded in Lebanon in 1993, Yemen in 1995, United Arab Emirates in 2000, Bahrain in 1993 and again in 2002–2003, Oman in 2009 and finally in Saudi Arabia and Iraq in 2013 [117,118].

Sporadic occurrences of LSD in several European countries (Greece, Albania, Russian Federation) were first recorded in 2015. A massive outbreak was noted in Russia in 2017 and cases were recorded in several countries within the European continent, where it was mostly successfully controlled with mass vaccination and appropriate managerial practices [26]. Although Georgia started vaccination of cattle in 2014 with the outbreak of neighboring Azerbaijan, an outbreak was recorded in 2016 and re-emerged with 6 more outbreaks in 2018 [119]. At the same time, LSD outbreaks were reported in eight Balkan countries (Greece, Bulgaria, The Former Yugoslav Republic of Macedonia [FYROM], Serbia, Kosovo, and Albania) with higher incidence at the border areas [120].

**Figure 2:**
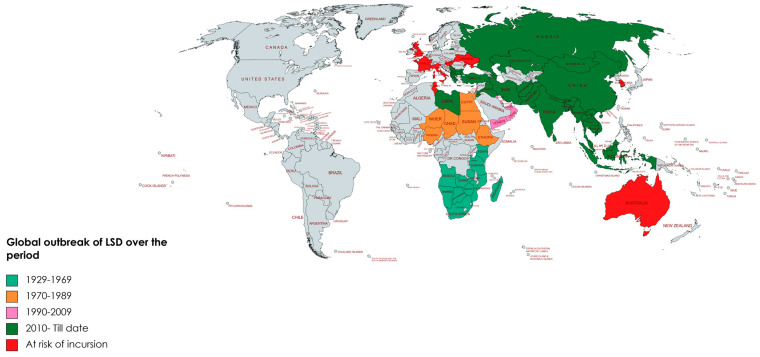
Global outbreak of natural lumpy skin disease (LSD) starting from 1929 to 2023?

Since 2019, LSD has become a major concern for Asian countries and presents one of the greatest threats for animal health and food security on the Asian continent. The reports of the devastating LSD outbreak in Bangladesh in July 2019 emphasized LSD’s importance [23,121] and this was again evident with a subsequent outbreak in 2020 [76]. Subsequently, many outbreaks have been reported across Asia, including simultaneous outbreaks in China and India [13,44]. The intra-country spread of LSD has been extensive and rapid with frequent outbreaks now occurring on a regular basis [122]. Outbreaks have been reported in Nepal, Bhutan, Sri Lanka, Vietnam and Malaysia in 2020 at the month of June, July, September, October and November, respectively [44,81]. An outbreak in Thailand was reported to WOAH in April 2021, subsequently also in Lao People’s Democratic Republic in May and in Pakistan in November of the same year [11,13,123]. Very recently, LSD was confirmed in Indonesia and Singapore in March 2022, [124]. The emergence and re-emergence of LSD is a matter of great concern, especially since recent outbreaks have not followed distinct patterns previously reported [2]. Table 1 and Figure 2 present the historical occurrences of LSD and an indication of countries at threat of a possible incursion of this disease.

## 4. Diagnosis and Management

### 4.1. Presumptive Diagnosis

Primarily the clinical history, clinical signs, morbidity and mortality rate can provide some basis for the presumption of LSD infection. A confirmatory diagnosis of LSD is indicated in all cases where owners report cattle with characteristic necrotic skin nodules (sit-fast) observed on face, eyelid, neck, muzzle, nostrils, udder, limbs, enlarged lymph nodes, persistent high fever and gradual emaciation [13,164].

The clinical signs can occasionally be confused with other skin lesion producing diseases, requiring laboratory confirmation. Postmortem diagnosis is also important. In sum, pock-like lesions are found on the mucous membrane of the mouth and different parts of the alimentary tract, the nasal cavity, trachea and lungs [13,99]. The lesions may also be present in the testes and urinary bladder [13,70]. Severe oedema is found in the dependent parts of the body with enlarged mediastinal lymph nodes. Synovitis and tenosynovitis with fibrin formation are detected in the synovial fluid [165]. Skin sample from affected animals may be useful for diagnosis on histopathology [37]. Eosinophilic intracytoplasmic inclusion bodies marked in the keratinocytes, macrophages, endothelial cells and pericytes from skin nodules is considered a pathognomonic lesion, along with ballooning degeneration of the epidermal cell layers [166]. Other inflammatory cells such as macrophages, lymphocytes and eosinophils are also infiltrated in the affected area. Widespread vasculitis, thrombosis, infarction, perivascular fibroplasia are seen histologically [104]. Due to muscular involvement, sever coagulative necrosis (Zenker’s necrosis) in subcutaneous muscle may be observed histologically. Lymph node enlargement is associated with lymphoid proliferation, oedema, congestion, and hemorrhage [9,102].

### 4.2. Confirmatory Diagnosis

The skin biopsy sample is an excellent source of virus for confirmatory test including virus isolation and identification. Where available, the samples should be collected and transported to the laboratory by using virus transport medium (VTM) such as 20 to 50% glycerol in phosphate buffer saline. Several laboratory diagnostic tests are recommended for the viral agent diagnosis such as, virus isolation, conventional or real-time polymerase chain reaction (PCR) and electron microscopy. Several serological tests such as virus neutralization (VN), agar gel immunodiffusion (AGID), indirect fluorescent antibody test (IFAT), Western blot analysis and enzyme-linked immunosorbent assay (ELISA) are available for detection of immune response of infected animal specific to LSDV [13,37].

Both conventional gel-based PCR methods and quantitative real-time PCR methods are considered as rapid, simple and sensitive tests, whereas RT-PCR is considered more sensitive, faster and labor-saving than conventional PCR [160,167]. PCR is the most popular testing method for the detection of viral genome from skin lesions (scabs or nodules), un-clotted blood, saliva, nasal swabs, semen, milk, and tissue culture samples following the standard protocol specific for capripoxvirus [37,168].

LSDV isolation and identification in cell/tissue culture is well established. Tissue cultures of bovine, ovine or caprine origin, especially primary or secondary culture of bovine dermis cells, kidney cells (Madin–Darby Bovine Kidney, MDBK) cells, lamb testis (LT) cells, etc., are considered to be the most susceptible cells for LSD viral growth. The chorioallantoic membrane of embryonated chicken eggs and African green monkey kidney (Vero) cells are also suitable for LSDV growth [37,169].

Electron microscopy can be used to identify the classic poxvirus virion by using a negative staining preparation technique. Brick shaped, covered in short tubular elements and approximately 290 × 270 nm in sized capripox virion are well observed with pioloform-carbon substrate under electron microscope. This test method is, however, limited in its ability to distinguish LSDV from other orthopox species or varieties [13,37].

Immune responses of either infected or recovered animals can be detected by different serological tests; however, some tests are unable to distinguish LSDV from other species of capripoxvirus [37]. Detection of antibody in infected or recovered cattle, using virus neutralization test (VNT), is successful between 2 days post-infection and approximately 7 months after infection. VNT has a limitation of antibody detection with low titers and its sensitivity has been reported to be approximately 70% [48]. An indirect fluorescence antibody test (IFAT), using the capripoxvirus antigen fixed in the tissue culture plate, can be used to detect antibody titers of up to 1/5000 in the serum of convalescent animals [26]. It has the capacity to test a larger number of samples than VNT. However, it has a limitation of cross-reaction with cowpox virus, but not with parapox viruses [8]. Agar gel immunodiffusion (AGID) test is a simple and cost effective procedure used for detection of precipitated antigenic particle but this method is not recommended for LSDV [37] due to cross-reaction of LSDV with antibodies to bovine papular stomatitis and pseudocowpox virus resulting in false-positive results [64].

Western blot analysis is designed with sensitive and more specific configuration for detection of capripoxvirus antibody but this method is time-consuming and expensive due to its requirement of pure antigen [37]. A new commercial and WOAH recommended enzyme-linked immunosorbent assay (ELISA) kit is commercially available for the detection of blood antibody against LSDV. Experimentally, ELISA has high specificity and does not cross-react with parapox viruses, but it cannot differentiate between antibodies against LSDV, SPPV and GTPV virus in serum and plasma [164]. Immunohistochemistry investigations can demonstrate LSDV antigen particles within the cytoplasm of epidermal basal cell layer, especially in prickle cells; the reactions demonstrated as a granular golden brown immunoperoxidase staining of viral antigen [170].

### 4.3. Differential Diagnosis

Since there are couple of other diseases with similar skin lesions to LSD, the differential diagnosis is important [99]. Pseudo lumpy skin disease is caused by bovid herpesvirus 2 (BHV2), which causes superficial skin lesion and is characterized by a short course of disease. Pseudo cowpox (Para poxvirus) is differentiated from LSD lesions by its sites of infection, which is mainly on the teat and udder. Vaccinia virus and Cowpox virus (Ortho poxviruses) also cause site-specific lesions on the teat, udder and muzzle and are zoonotic in nature, unlike LSD. Dermatophilosis caused by *Dermatophillus congolensis* and *Hypoderma bovis* infection may cause confusion due to its similarities with LSD. However, the lesions are severely swollen and eroded, with exposure of larvae in the skin of the back of the animal. There may be paralysis of the lower body and legs when the spinal cord is involved. Onchocercosis and demidocosis are parasitic skin problems often characterized by the presence of parasites and they are usually site specific. There are other hypersensitivity reactions (photosensitization, insects, and arachnid biting), which may create confusion with LSD lesions. The differentiation of these diseases can be done through PCR and other antigen or antibody specific tests [39,171].

### 4.4. Treatment Strategies

Similar to other viral diseases, there is no definite treatment protocol for LSD apart from symptomatic [84] and supportive treatment. Treatment may include antibiotics for the secondary bacterial infection, anti-inflammatory pain-killer therapy to reduce pain [60] and local application of ointment or antiseptic spray for wound healing [72]. Often intravenous fluid therapy is used to address dehydration and vitamin supplementation is given to reduce the weakness and increase the appetite [110]. It is worth mentioning that autohemotherapy and autogenous serum therapy from a recently recovered animal to the infected animal is being practiced in different areas of Bangladesh following Bangladesh Livestock Research Institution (BLRI) guidelines as a trial (Mahmud T., Personal communication, 7 July 2023). However, proper scientific research is yet to be performed, and no further data are available.

## 5. Vaccines and Vaccine Controversies

According to the WOAH, appropriate vaccination can control LSD by providing good immunity to cattle against LSDV. The vaccines should be safe to use for all cattle breeds, ages and pregnant animals and the vaccines should be well prepared with an appropriate label. Recently, the use of live attenuated vaccines of both homologous (vaccinating cattle with a LSDV-based vaccine) and heterologous (vaccinating cattle using a sheep pox/goat pox virus-based vaccine) has been used to control the outbreak in endemic regions [18]. The heterologous vaccines are used as alternative options in those areas, where both LSD and sheep and goat pox occur simultaneously, and the countries have manufacturing capacity for these vaccines [10,18]. However, the heterologous vaccines should be well characterized, adjusted, and evaluated using a vaccine challenge trial.

The available attenuated vaccine consists of either the well-known South African Neethling strain or the Kenyan sheep and goat pox (KSGP) O-240 and O-180 strains. The vaccine-producing viruses are derived from 61 serial passages in lamb kidney (LK) cells culture, followed by 20 passages in the chorioallantoic membrane of embryonated chicken eggs, and three passages again in LK cells [172,173]. The South African Neethling strain is the vaccine most commonly used in endemic countries to protect cattle against LSD. It has been shown to be effective; however, it can cause some adverse effects. It has been reported that vaccination with Neethling strain may cause local skin reaction at the vaccination site or generalized small sized skin nodules with a reduction in the milk yield from lactating cows, often referred to as “Neethling response” [63,99,172]. Several studies have reported that vaccine viruses were detected from skin nodules, blood, and milk of cattle vaccinated with Neethling vaccines [174]. The KSGP O-240 and O-180 have been used against LSD in the horn of Africa, Israel and Egypt, and reported to give protection with post-vaccination complications such as fever and skin lesions [100,175,176]. Inadequate protection with comparatively higher morbidity rate were reported in dairy cattle vaccinated with both KSGP O-240 and KSGP O-80 strains [174]. Another example of attenuation of a virulent field strain is the Madagascan LSD strain. The original LSDV strain is also reported to be used for controlling LSDV [177].

The Gorgan goatpox strain (a goatpox virus-based vaccine), Romanian SPPV strain (a sheeppox virus-based vaccine), and the Yugoslavian SPPV RM65 strain (Ramyar) are used as heterologous vaccines against LSDV. Gorgan goatpox strain vaccine was first used against LSD Ethiopian cattle and reported to give sufficient protection and seroconversion in cattle against highly virulent LSDV [17,178]. The immunogenicity and immune response of heterologous vaccines are well studied compared to that of the homologous formation [17,110,179]. A minimal, or in most cases, no adverse reaction was found at the injection site [18]. Varying degrees of success was reported with the use of different heterogenous-type sheeppox based vaccine in the Russian Federation, parts of the Middle East, and Africa [180]. These vaccines were demonstrated to afford lower protection in cattle against LSDV and some effects such as fever, reduced milk production, the development of nodules in vaccinated cattle, and sometimes re-emergence were also recorded in the vaccinated herd [18,134]. Despite minimal adverse reactions, the heterologous vaccines usually require a lower level of attenuation for safe use in cattle than homologous vaccines [81]. The production cost and price of homologous vaccines are also much higher because of the requirement of a higher number of passages for attenuation in comparison to the heterologous products [168].

Inactivated LSDV vaccines are not yet described in the WOAH Manual, but some manufacturers have developed inactivated vaccines against LSD for countries that may be willing to use it [18]. Inactivated vaccines generate a shorter duration of immunity and may be preferred as a prophylactic vaccine alternative in disease free, at-risk countries [18,181]. Nevertheless, recombination might be possible due to improper safety maintenance during the manufacturing process. Recombinant LSDV could be developed when under-attenuated vaccines are introduced in animals previously infected with virulent field strain [182,183]. The risk of cross-contamination is another threat to consider when using vaccines of different strains or multivalent vaccines of the same species [180,184,185]. Sometimes emergence of multi strain LSDV could result from improper safety measures during the propagation of viruses [186]; recombination may also occur [187]. Appropriate molecular tools should be available for screening of viral strains present in the LSDV vaccine, which could minimize the risk of recombination and cross-contamination [18]. Some studies have suggested the need to distinguish between the recombinant wild-type LSDV strains and the Neethling-based vaccine strains which are used in most live attenuated commercial vaccines [188].

Vaccination should be done as per manufacturer instructions, but some common instruction must be maintained for effective vaccination programs. Annual vaccination of adult cattle, calves from vaccinated animals, or newly purchased animals before entering a property, is recommended [177]. However, animals which might be in their incubation period or actively infected, should strictly not be vaccinated with live attenuated vaccine as there is a risk of recombination of virus and vaccine strains [189]. In the case of inactivated vaccination, initial vaccinations comprise two vaccinations one month apart and then re-vaccination every six months is required to maintain immunity [182].

## 6. Economic Impact

LSD has both direct (related to mortality) and indirect (related to impacts of the disease on animal health and production) impacts on countries’ economies, with indirect effects outweighing direct effects [16]. The mortality rate of LSD is low and usually considered as 1–3%, whereas the morbidity rate varies from 3% to 85% worldwide [44]. The major economic losses are followed by the high morbidity rate along with subsequent disability of the infected animals [15], costly treatment and control measures, restricted global animal trade and higher cost of maintenance of proper biosecurity measures [44]. Chronic effects found in both dairy and beef cattle include emaciation, cachexia, permanent scar formation in the skin lesion resulting in decreased hide and meat quality [14,15] and reduced draft power of oxen used in mixed farming systems [16] in some parts of the world. Loss of production in cows include a decline in milk yield due to high fever, development of secondary bacterial mastitis and abortion [48,100]. In bulls, severe orchitis cause temporary infertility that may lead to permanent infertility, if severe [17,51]. To mitigate the clinical signs, symptomatic treatment measures should be evaluated from a financial perspective. Often, infected or in-contact animals are slaughtered for eradication and control of the disease. Strict biosecurity measures are also adopted, which become an economic burden [44,190]. All these factors lead to reduced quantity and quality of production and are associated with the trade restriction of animals and animal products resulting in economic losses for the industries and countries involved with livestock [10,104]. Lumpy skin disease is currently exerting a huge economic impact on the livestock industry as the disease is wiping out small scale marginal farmers. For example, a total annual loss of approximately 31.37 and 59.97 million USD were recorded from two districts (Gaibandha and Mymensingh) of Bangladesh [191]. The incursion of LSD in LSD-free countries could have devastating economic consequences. It has been estimated that LSD incursion into Australia could cost millions of dollars [192].

## 7. Transboundary Biosecurity Threat

Lumpy skin disease is categorized as an important transboundary disease and several factors are associated with the rapid cross-border spread of LSD [189,193]. Legal or illegal transportation of both live domestic and wild animals, including their products, (milk, meat, hides and skin and biomaterials such as embryo, semen, blood and bone), all present threats for both short and long-distance transmission of LSD [182,189]. Vectors are an important factor to consider in the transmission of LSDV, especially over longer distances. Vector abundance in the border area is also associated with favorable.

Environmental conditions such as temperature, humidity, wind and season of the particular area [64]. Live animal movement from the infected area to the at-risk area pose a significant threat [194], as does unpasteurized milk from infected and carrier animals imported from the threatened zone to free or at-risk zones [195,196]. Trade of hides, skin, wool and other fiber, different animal byproducts, animal wastage and effluents are also important considerations in transmission of LSDV [36]. Some biomedical components from infected animals such as embryos, semen, dried blood, bone and even whole carcass may also be implicated in transmitting LSDV [64,70,189]. Equipment which are involved in the transportation of animals and their products from contaminated areas, as well as animal handlers, are also considered to be threats for the transboundary transmission [195,197]. Vaccinated animals may sometimes pose a threat if they experience activation of live attenuated virus, so the probability of infection by the transportation of vaccinated animals should not be ignored [182,189].

## 8. Biosecurity Policies

Robust biosecurity policies are pivotal to control and prevent LSD. Control measures in endemic areas are largely dependent upon vaccination, movement restriction of infected animals and vector control, but incursion of LSD can be avoided by strict animal movement control from the infected countries or areas and through maintenance of proper biosecurity measures at the farm level [20]. Biosecurity policies for controlling and eradicating the incursion include proper management of input products, management of production practices, movement management of humans, vehicles and other equipment and finally, through management of vectors [198]. The main input materials of a farm include the cattle, feed and water and the bedding material. Proper biosecurity measures should be maintained during entrance of such products to minimize the risk of infection. Cattle purchases should be done from preferred suppliers and quarantine should be maintained before introduction to the feedlot [135,198]. Ensuring the quality of feed materials and safe and suitable water supply is a key point in mitigating against any kind of infection and essential to improve immunity. Production practices include monitoring of the infected animals, management of manure, effluents and carcasses which could be a further source of contamination [20]. Routine monitoring of sick cattle and disposal of different materials and carcasses should be mandatory with proper hygienic measures for effective control of this disease [171]. To minimize the risk of introduction and spread of disease or contaminants, personnel including employees, family, visitors, service personnel and veterinarians should use personal protective equipment (PPE) when attending the animals or for the disposal of carcasses [145]. There should be minimal movement of necessary equipment and vehicles to prevent the introduction of LSDV into the shed. Strict and well-defined policies for proper cleaning of vehicles, shoes, farm machineries and equipment should be in place [99]. Control of insect vectors is of outmost importance to control an outbreak of LSD because insect vector is the mechanical transmitter of LSDV. Limiting vector breeding sites such as standing water, slurry, or manure by improving drainage system is important. Various insecticides can control breeding of LSDV vectors such as flies, mosquitoes, ticks and midges. Use of chemical agents and insect repellents in the infected premises and on the skin of livestock will reduce mechanical transmission of LSD [145]. Apart from biosecurity measures, proper tracing and surveillance is required to map and zone the possible risk area. Regular awareness campaigns among veterinarians, students, farmers, herdsmen, cattle traders, cattle truck drivers and artificial inseminators, and vaccination with inactivated vaccines could be adopted to minimize possible threats of an LSD outbreak [195]. In addition to farm biosecurity measures, a coordinated biosecurity and one health effort of Local Government, state and Federal Government and, most importantly, regional and global Government, non-governmental organizations (NGO) and industry efforts are warranted.

## 9. Incursion Threat and Global Chaos

Continuous emergence and re-emergence of LSD in different countries makes it a great livestock health concern. Since LSD is a transboundary disease, due to increased trade demand of animal and products, a possible threat of intrusion is becoming alarming for LSD-free countries. Countries with no record of LSD infection such as Australia, New Zealand, United Kingdom (UK), USA, France, Italy, Belgium, Hungary, Spain, Portugal, Slovenia, Slovakia, Croatia, Bosnia and Ukraine [199] are bearing a high risk of disease incursion associated with shared borders with countries with outbreaks. In countries where outbreaks have been managed, recurrent emergence is a risk should control and prevention methods be less stringently applied. Kenya, Romania, Russia, Moldova, Georgia, Armenia, Azerbaijan, Tajikistan, Mongolia, Turkey, Israel, Iraq, Syria, Jordan, Afghanistan, Pakistan, India, Nepal, Bangladesh, Sri Lanka, Myanmar, Thailand, Malaysia, Singapore, China and Indonesia have all recently been faced with re-emergence of LSD with severe animal health and economic effects [196].

A recent outbreak of LSD in Libya is an obvious example of incursive nature of LSDV. It is likely that the source of this outbreak was the importation of infected animals, indicating transboundary transmission [155]. Transboundary incursion may be resulted from either the mechanical vector borne transmission for short distance spread or by the movement of infected animal and infected vehicle for long distance spread [200]. Latest outbreak of LSDV is reported by the Indonesian Government in the Sumatra Island and Bali of Indonesia which increases the risk for introduction of LSD in Australia because of Australian tourists regularly visiting Bali [201]. Similarly, the outbreaks in Turkey represent a threat for the neighboring EU countries, especially Greece and Bulgaria, which can be at risk of new incursion. The cost of an incursion would produce severe economic losses due to stock losses and reduced production, including reduced milk yield, loss of animal body condition and rejection or reduced value of the hide. Hence, lack of regional and international collaboration could lead to a worldwide chaos in the livestock industry. LSD-free countries should be in high alert and take necessary coordinated actions to protect the livestock industry. For example, Australia does not import live cattle or their germplasm from LSD-infected countries. The chance of arthropod vectors entering via aircraft is low, but strong border managements are in place for in bound travelers in Australia [200]. Furthermore, a National Lumpy Skin Disease Action Plan aimed to improve Australia’s preparedness for a potential incursion of LSD is designed by the Minister for Agriculture, Fisheries and Forestry, Australia [12]. Minimizing the spread of the disease through early detection and reporting will reduce the economic and social costs of an outbreak to livestock producers, regional industries and national economy of possible threatened countries [183]. A systemic review has been recently published on LSD transmission and risk of emerging [202]. This review has extensively data mined and listed research articles published on LSD and has summarized different experimental studies. However, this review is critically discussing the historical outbreaks, genomic resources, limitations of vaccinations and diagnosis, and importance of international collaborations to mitigate the global threats of LSDV.

## 10. Conclusions

LSD is a global concern to the livestock industry due to its rapid spread in recent years accompanied by the reported huge economic impacts. There is a global threat of continued spread of the disease as observed in Africa, the Middle East and Asia, and it currently threatens the Western Europe and Australian livestock industries. Due to its fast-expanding nature, huge livestock losses would be inexorable. Proper vaccination, vector control, restriction on importing animals, and animal products from affected countries, quarantine measures, disease surveillance programs, quick and early diagnosis of infectious agent with typing, and stamping-out of infection would be possible measures for controlling the emergence and preventing the re-emergence and incursion of LSD. Moreover, proper disposal of carcasses and infected materials, disinfection of affected sites and strict movement control should also be followed for complete protection. There is a need for collaborative and global action towards the control and eradication of LSD in different parts of the world. As vaccination is the only way to prevent the emergence, re-emergence as well as incursion of this disease, strategies such as vaccine development are deemed important, informed by high-throughput LSD surveillance and LSDV genomic data across the incursions. LSDV genomic data will allow for accurate identification of the potential incursion pathways, which is crucial for devising effective biosecurity measures to keep LSDV at the bay. Furthermore, the genomic data will pinpoint any emerging LSDV variants, thus informing the efficacy of the existing LSDV vaccines and revealing new target sites for vaccine optimization if required.

## Figures and Tables

**Table 1:** Historical outbreak of natural lumpy skin disease (LSD).

Year	Country	Probable Origin	Strain, Vaccine and Vaccine Complication	Brief Epidemiological Data Including Economic Importance	Re-Emergence and Possibilities of Re-Emerging	Global Threat	Reference
1929	Zambia	Not clear	Limited data on strain Insufficient vaccination	Acute, Subacute or inapparent Countrywide distribution (Eastern, Northern and Central province)	33 outbreaks occur in 1987	Other African countries	[125,126]
1943	Botswana	Zambia	Neethling strain, controlled through vaccination with live attenuated vaccines (LSDV Neethling strain) and South African LSDV field isolate (Lumpyvax)	Named as ‘Ngamiland Cattle Disease’ Endemic type, transmission occurred by biting arthropods	Outbreaks occur in almost every year since 1943	Zimbabwe and South Africa	[109]
1944–1945	Zimbabwe	Zambia and Botswana	Neethling virus, live attenuated virus vaccine Neethling strain: immunity conferred lasts up to 3 years	Cattle movement from communal areas into the previous commercial farms. After land reform program launched in 2000, outbreaks occurred on large-scale commercial cattle farms	Re-emergence occurs almost every year	Infected animals exporting countries	[127,128]
	South Africa		Neethling-type strain Live attenuated South African LSDV field isolate	Known As ‘knopvelsiekte’, transportation of cattle is the possible route of entry and biting flies (*Stomoxys calcitrans* and *Musca confiscate*) are the transmitting vector. Approximately 8 million cattle were affected and massive economic loss due to reduced production	Severe outbreak occurred in 1953–1954 and epizootics continued to 1968	Sudan and Ethiopia	[129,130]
1946–1956	Mozambique, Angola, Madagascar, Namibia, Tanzania and Uganda	Zambia and Botswana	Neethling-type strain Limited available data of vaccination	Outbreak spreads slowly and erratically Cattle transportation is the mode of transmission. No detailed information is available	Re-emergence in almost every year with minimal outbreak	Other cattle transporting, border-side countries	[131]
1957	Kenya	No record found in online search	LSD/240 strain of lumpy skin disease virus, identical with the Neethling strain Vaccine derived from Kedong Valley strain of sheep pox virus	Primarily epizootic in nature but subsequently sporadic, vector borne infection persists in the areas of high-altitude indigenous forest in a fairly high rainfall zone with very lower morbidity of 1–2% but initiate considerable economic losses by decreasing production performances	Re-emergence in almost every year with minimal outbreak	Several African territories by entry of exotic breed	[8,110]
1971	Sudan	Limited data available	Neethling-type strain Live attenuated local strain of LSD used as vaccine	Epizootic in nature and later become endemic of a milder form for local breed but fatal for exotic, transmitted mainly by biting arthropods (Tick, *Amblyomma* spp.) and aerosol transmission, affect mostly larger dairy farm and usually after rainy season	several outbreaks in Khartoum, Gezira and the River Nile States during the period 2004–2006	Limited data available	[111,112,131]
1973–1974	Cand, Niger and Nigeria	Cameroon through Gongola State (Nigeria)	LSDV V/281-Nigeria field strain Live attenuated homologous and heterologous vaccines were used	Epizootic initially with gradual turn into endemic pattern, outbreaks occurred in rainy season and spread by combined effect of wind and movement of host and vector with very insignificant direct loss but persistent economic losses was recorded	In 1979–1980 more severe and widespread outbreaks were recorded	Limited data available	[113,114]
1981–1983	Ethiopia	Sudan	Neethling virus prototype strain Live attenuated Kenyan sheep and goat pox (KSGP 0–180) vaccines produced effective control approach	It is epizootic in almost all the regions and agro-ecological zones of Ethiopia, highly associated with climatic conditions, mainly heavy rainfall, favoring the increased vector population (Biting insects), sero-prevalence was higher in the midland agro-climate zones. The financial losses reflect the loss both animal and animal products	Major epidemic outbreaks were in 2000–2001 in and epidemics recorded up to 2010.	Limited data available	[35,49,115]
1988–1989	Egypt	Africa	Similar to the African, Asian and European strains A heterologous vaccine (Romanian sheep pox vaccine) provided sufficient levels of protection.	Is typical exotic disease, usually entered through importation of live animals. Within a few short years, it transformed into enzootic status, arthropod vectors are main source of infection and the activity of vector increased in the wet weather of rainy season	Re-emerged with enzootic nature in 2006, 2011 and 2014 and going on with endemic nature	European countries and specially Israel	[72,117,132]
1889	Israel	Egypt	No reported data found about strain. Vaccination was failed with Yugoslavian RM 65 sheep pox strain vaccine, but effectiveness was derived by repeated vaccination with RM 65 sheep pox strain vaccine	Epizootics of this disease were associated with high humidity with warm and moist condition and possibly spread by stable flies (*Stomoxys calcitrans*), charecteristics with mild clinical form and lower morbidity and mortality	Outbreaks occurred in Israel in 1989, 2006, 2007, and 2012 subsequently.	Limited data available	[41,133]
1990–2010	Middle eastern countries #	Egypt and Israel	Neethling-type strain Unclear data about the maintenance during inter-epidemic periods but proper vaccination, strict biosecurity and slaughter policies were noted for eradication	This disease was epizootic in nature in all the countries, considered as the transboundary transmitted mainly, having a significant effect on trade and food security and there was much variation in morbidity, mortality and disease spread rate	Several occurrences sporadically with the cross-boundary transmission	All the surrounding countries were subjected to be pandemic exploration	[50]
2012–2014	Middle eastern countries ##	Syria and Iraq	Neethling-type strain, Vaccination with Bakirkoy sheep pox strain, RM-65 sheep pox strain and the unlabeled LSD strain specially in Jordan were used for control	Primarily an epizootic disease having a risk of being endemic in each country, without or with a very low abundance of arthropod vectors but was likely to be associated with the illegal movement of clinically sick or asymptomatic infected animals and vectors	Several occurrences were recorded in different provinces of individual country	Greece and Bulgaria	[134,135]
2015–2017	Russia	Turkey, Azerbaijan, Iran, and Kazakhstan	Neethling-type strain, A heterologous (SGPV strain vaccine) has been used with a coverage of approximately 70% protection	Epizootics nature was responsible for significant damage with high morbidity and low mortality rates, usually spread occurred by illegal, infected animal movement, had a severe attack with approximately 7% infected animals died	Subsequent re-emergence in following years up to 2019	Northern regions of Europe	[136,137]
	Balkan Countries ###	Turkey	Neethling-type strain, A live attenuated homologous vaccine (Neethling strain and SIS Neethling type) was used and reported a better immunity	This epidemic had a strong seasonal pattern, with a summer peak and a winter drop, along with a abundance of arthropod vector resulting a faster spread of over 7600 LSD outbreaks with approximately 12,800 affected animals were reported in 2015	No outbreaks were reported in 2018	Central and South Asian countries	[39,121,138]
2019	Bangladesh	Unresolved but may be from neighboring countries	LSDV are 99.99% homologous with two old African field strains-Neethling 2490 and KSGP 0–240 Prophylactic vaccination with attenuated LSD or goat pox viruses are being practicing	An emerging threat to cattle health with major socioeconomic impact by production losses, added treatment costs, chronic debilitation and death of the animals, massively influenced by the geographical distribution and seasonal pattern and mechanical transmission by arthropod vector	Re-emergence was found in 2020 with huge high morbidity and mortality was also recorded.	India, Myanmar	[23,76,121]
	India		LSDV were very closely related to the Neethling NI-2490/1958, Kenya/1958 and KSGP-like strains. Live attenuated vaccines of capripoxvirus (Kenyan sheep and goat pox strain (KS-1), Yugoslavian RM-65 sheep pox strain, Romanian sheep pox strain and South African strain) are currently being using	Endemic occurrence was carried out by mechanical arthropod vector (mosquitoes, biting flies, Culicoides, midges and blood sucking hard ticks) and vector abundance is influenced by wet and warmer condition of summer and autumn months, causes approximately 2 hundred thousand cattle infection with death of approximately 97,000 cattle resulting severe economic losses by the means of individual death and reduced milk yield of 20% till 2022	Outbreaks are ongoing in different states of India	Nepal and Bhutan	[139,140,141]
	China	Kazakhstan and Russia	Three strains were found (LSDV/China/XJ01/2019 China/GD01/2020 and LSDV/Hongkong/2021) and related to the Neethling strain. Live attenuated goat pox vaccines are used	The epidemic nature outbreak occurs in different provinces and believed to be spread through the arthropod vector and uncontrol cattle and animal products movement, caused a devastation for the cattle industry. The morbidity and mortality of each outbreaks ranged 6.6–100% and 0–16.7%, respectively	LSD was spread to Southeast China in 2020 and caused outbreaks in multiple provinces	Taiwan	[142,143,144]
2020	Nepal	India and China	LSDV NI-2490 strain is identical to the strain isolated from Kenya, Bangladesh and India Lack of available vaccines Prevention and control is maintained by proper biosecurity, vector control, movement restriction and treatment of diseased animals	This re-emerging disease can spread rapidly during summer and autumn months, in moist and warm environment (favorable for the growth and reproduction of houseflies, mosquitoes, etc.). The outbreaks are considered to be caused mainly by mechanical vector transmission. Along with 3–7% morbidity rate it can causes great economic losses by restricted animal products to the global trade and costly control and eradication measures	Regular outbreaks are recorded till to date	Data not found	[145,146,147,148]
	Vietnam	China	LSDV of the Vietnamese LSD samples shows a similarity with Neethling virus strains and an identity with Chinese and Russian LSD strains. Both homologues (Neethling strain) and heterologous (Gorgan strain and RM65 strain) vaccines are the possible controlling option	This easily spreading disease spread mainly through insect bites such as mosquitoes, flies, ticks and can also be transmitted through transport of pathogen carrying animals and then share drinkers, feeding areas, milk, semen with others. Although morbidity was recorded approximately 10–20% but the economic effects were not properly mentioned	A total of 93 LSD outbreaks were reported in 93 communes of 36 districts.	Data not available	[149,150,151]
2021	Thailand	Not mentioned	The Thailand strain is similar to the China/GD01/2020 and Hong Kong/2020 isolates. Live attenuated Neethling LSD vaccines have been disseminated for disease control	The emergence was supposed to get entry through illegal movements of infected and carrier animals from the source country and possible inter-farm transmission was occur with the insect vectors such as stable flies and mosquitoes. The overall morbidity and mortality rates were 40.5% and 1.2%, respectively	Several outbreaks were recorded in different provinces till date	Data not available	[152,153,154]
	Pakistan	India	LSDV strains have shared the highest genomic homology with strains reported from India, China, and Bangladesh No successful vaccination protocol was found	LSD was noted as most dangerous, devastating, endemic disease, transmitted by transboundary transmission with allowing the infected population and vectors were observed in all the affected farms It causes serious economic implications by reduced milk production, infertility in cows and bulls, emaciation, abortion, skin damage and death	Still, it is the major threat for livestock in Pakistan because of its rapid spreading nature	Not clearly mentioned	[10,154,155,156]
	Mongolia	Russia and China	Mongolian isolates shared 100% identity to Chinese, Vietnamese, Russian, and Kazakhstan isolates. No vaccination was reported, only control and preventive measures were adopted	The clinical prevalence of LSD in cattle was approximately 6%, that leads a huge economic loss including restrictions on international trade of live animals and losses animal products such as milk, meat, and hide.	Data not available	China and India	[157]
	Cambodia		Strain data are not clearly reported but collaborative vaccination to domestic livestock was performed with Lumpyvax TM	Not clearly mentioned but continuous outbreak was recorded in different parts of the country			[158]
2022	Afghanistan	Unknown or inconclusive	Not reported in detail Control measures were carried out at the event level with ante and postmortem inspections, vectors control, movement control, selective killing and disposal, slaughtering, surveillance within and outside the restricted zone	Limited data available			[159,160]
	Korea	China and Nepal	A serological and molecular prevalence study of LSDV in Korean water deer, native and dairy cattle was performed in South Korea, but no positive reactor was detected. No data is available about LSDV in North Korea.	Limited data available			[161]
	Indonesia	India	Limited data available. LSD has infected more than 22,000 animals in 13 provinces including Bali in Indonesia and the outbreaks continue. As Indonesia shares a border with northern Australia, it has become a great threat for the Australian cattle industry				[162]
2023	Libya	Unknown or inconclusive	Not detected yet and no information available yet on vaccination	Zonal infection was noted, 10 infected cases were recorded among 26 susceptible cattle with 3 death case	The infection may have already spread beyond the observed/recorded foci, either by vectors or by movements of animals	Tunisia, and other North-West African (Maghreb) countries	[163]

# = Kuwait, Lebanon, Yemen, United Arab Emirates, Bahrain, and Oman; ## = Jordan, Iraq, Turkey, Iran, Cyprus, Azerbaijan and Kazakhstan; ### = Greece, Bulgaria, The Former Yugoslav Republic of Macedonia [FYROM], Serbia, Kosovo, and Albania.

## Data Availability

Not applicable.
